# HLA-focused type 1 diabetes genetic risk prediction in populations of diverse ancestry

**DOI:** 10.1007/s00125-025-06563-8

**Published:** 2025-10-02

**Authors:** Dominika A. Michalek, Courtney Tern, Catherine C. Robertson, Wei-Min Chen, Suna Onengut-Gumuscu, Stephen S. Rich

**Affiliations:** 1 Department of Genome Sciences, University of Virginia, Charlottesville, VA USA; 2 Channing Division of Network Medicine, Brigham and Women’s Hospital, Boston, MA USA; 3 Department of Computational Medicine and Bioinformatics, University of Michigan, Ann Arbor, MI USA

**Keywords:** Genetic risk score, HLA, Multi-ancestry, Transferability, Type 1 diabetes

## Abstract

**Aims/hypothesis:**

Type 1 diabetes is characterised by the destruction of pancreatic beta cells. Genetic factors account for approximately 50% of the total risk, with variants in the HLA region contributing to half of this genetic risk. Research has historically focused on populations of European ancestry. We developed HLA-focused type 1 diabetes genetic risk scores (T1D GRS_HLA_) using SNPs or HLA alleles from four ancestry groups (admixed African [AFR; T1D GRS_HLA-AFR_], admixed American [AMR; T1D GRS_HLA-AMR_], European [EUR; T1D GRS_HLA-EUR_] and Finnish [FIN; T1D GRS_HLA-FIN_]). We also developed an across-ancestry GRS (ALL; T1D GRS_HLA-ALL_). We assessed the performance of the GRS in each population to determine the transferability of constructed scores.

**Methods:**

A total of 41,689 samples and 13,695 SNPs in the HLA region were genotyped, with HLA alleles imputed using the HLA-TAPAS multi-ethnic reference panel. Conditionally independent SNPs and HLA alleles associated with type 1 diabetes were identified in each population group to construct T1D GRS_HLA_ models. Generated T1D GRS_HLA_ models were used to predict HLA-focused type 1 diabetes genetic risk across four ancestry groups. The performance of each T1D GRS_HLA_ model was assessed using receiver operating characteristic (ROC) AUCs, and compared statistically.

**Results:**

Each T1D GRS_HLA_ model included a different number of conditionally independent HLA-region SNPs (AFR, *n*=5; AMR, *n*=3; EUR, *n*=38; FIN, *n*=6; ALL, *n*=36) and HLA alleles (AFR, *n*=6; AMR, *n*=5; EUR, *n*=40; FIN, *n*=8; ALL, *n*=41). The ROC AUC values for the T1D GRS_HLA_ from SNPs or HLA alleles were similar, and ranged from 0.73 (T1D GRS_HLA-allele-AMR_ applied to FIN) to 0.88 (T1D GRS_HLA-allele-EUR_ applied to EUR). The ROC AUC using the combined set of conditionally independent SNPs (T1D GRS_HLA-SNP-ALL_) or HLA alleles (T1D GRS_HLA-allele-ALL_) performed uniformly well across all ancestry groups, with values ranging from 0.82 to 0.88 for SNPs and 0.80 to 0.87 for HLA alleles.

**Conclusions/interpretation:**

T1D GRS_HLA_ models derived from SNPs performed equivalently to those derived from HLA alleles across ancestries. In addition, T1D GRS_HLA-SNP-ALL_ and GRS_HLA-allele-ALL_ models had consistently high ROC AUC values when applied across ancestry groups. Larger studies in more diverse populations are needed to better assess the transferability of T1D GRS_HLA_ across ancestries.

**Graphical Abstract:**

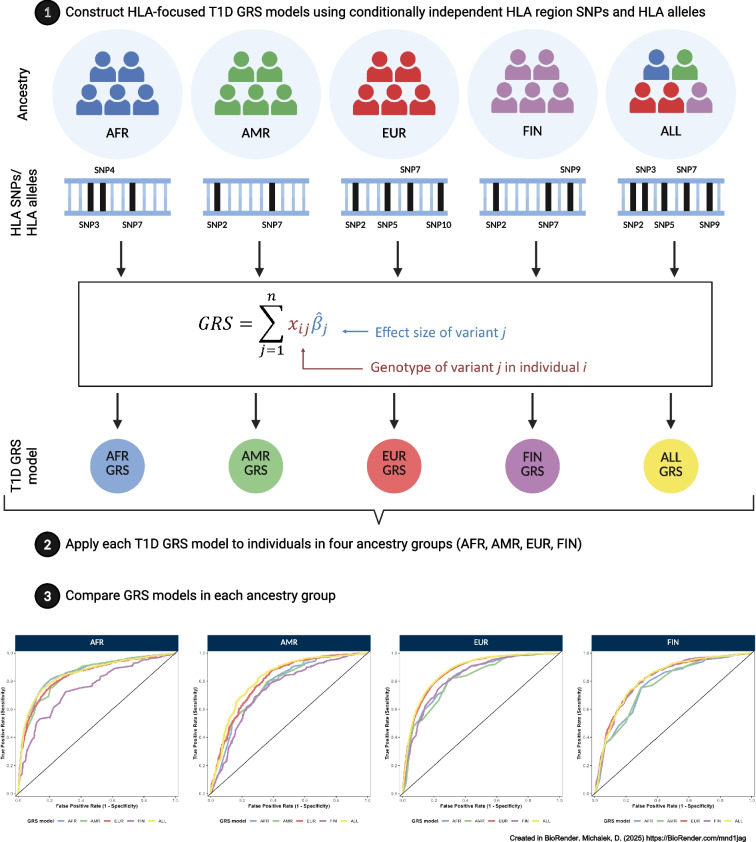

**Supplementary Information:**

The online version contains peer-reviewed but unedited supplementary material available at 10.1007/s00125-025-06563-8.


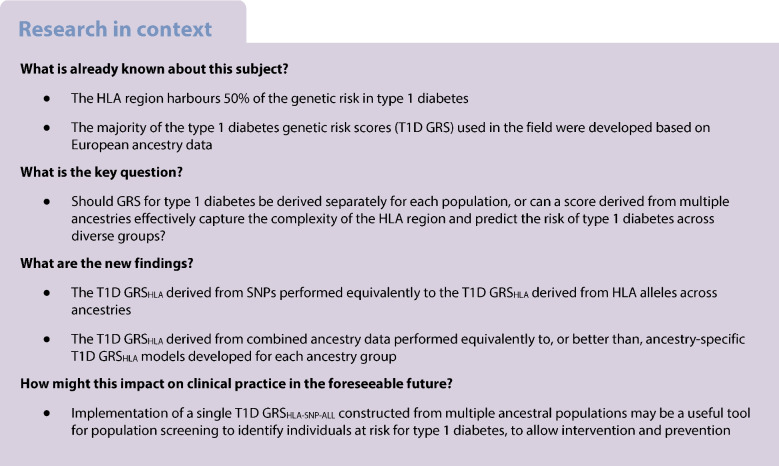


## Introduction

Genetic variation in HLA class I and HLA class II genes is integral to modulating immune responses [1, 2]. Numerous studies have identified a wide array of associations involving HLA alleles, with links to infectious diseases [3, 4], outcomes of organ transplantation [5], and risk of autoimmune diseases [6–8]. Type 1 diabetes results from autoimmune destruction of the insulin-producing beta cells in the pancreas [9], requiring exogenous insulin for survival. Twin and family studies have demonstrated that genetic factors contribute to approximately 50% of the risk for type 1 diabetes [10, 11], with variations in HLA class I (HLA-A, HLA-B, HLA-C) and HLA class II (HLA-DRB1, HLA-DQA1, HLA-DQB1) accounting for 30–50% of the genetic risk [12].

Epidemiological studies have suggested that the prevalence of type 1 diabetes in children is approximately 4 in 1000 in populations of European (EUR) ancestry [13], with the highest prevalence in Scandinavia (e.g. Finland [14]), as well as in Kuwait [15]. A lower prevalence in children of African (AFR), admixed American (AMR), South Asian (SAS) and East Asian (EAS) genetic ancestry has been observed; however, the SEARCH for Diabetes in Youth (SEARCH) study reported an increasing prevalence of type 1 diabetes in non-European ancestry populations in the USA [16].

The inclusion of non-European populations in genetic studies has been limited [17, 18], particularly for type 1 diabetes [19, 20], which is relatively rare in children of other genetic ancestries. More recent studies have expanded to include diverse populations, resulting in the identification of novel loci and new risk variants in known loci [21]; however, the role of HLA genetic variation in the risk of type 1 diabetes is high for European ancestry [22], although ancestry-specific HLA alleles and variants have been identified in non-European populations [21, 23–26].

A growing effort to utilise genetics in risk assessment has led to the development of genetic risk scores (GRS). Inclusion of genetic variants weighted by their impact on risk provides a single number that can be applied for individualised risk assessment. The initial type 1 diabetes GRS (T1D GRS) [27, 28] consisted of SNPs at both HLA and non-HLA sites in the genome. Many of the SNPs in the HLA region ‘tagged’ known HLA alleles associated with type 1 diabetes risk. Although several forms of the T1D GRS have been proposed, the T1D GRS1 exhibited good performance in classifying individuals at increased risk of type 1 diabetes, distinguishing risk of type 1 diabetes from risk of type 2 diabetes, and detecting those with type 2 diabetes who require insulin for glucose control [27]. The subsequent T1D GRS2 incorporated SNPs within the HLA region to estimate known heterozygote effects (e.g. HLA-DR3/DR4 increased risk) [28]. However, T1D GRS1 and T1D GRS2 are based primarily on data from EUR ancestry. Recent work demonstrated that an African American T1D GRS with seven SNPs outperformed the T1D GRS1 in AFR ancestry populations, and its performance was equivalent to that of the T1D GRS1 in populations of EUR ancestry [23]. There is a need to address the transferability of the impact of genetic variation in the HLA region on genetic risk in diverse ancestry groups.

In this study, we use dense genotyping in the HLA region in samples of EUR, AFR, AMR, and Finnish (FIN) collections to develop ancestry-specific T1D GRS_HLA_ and a combined T1D GRS_HLA_ based upon SNPs and their imputed HLA alleles. Each T1D GRS_HLA_ is applied to all populations to determine its performance in each ancestry group and to assess whether use of a combined (cosmopolitan) GRS or a series of ancestry-specific T1D GRS_HLA_ would be appropriate for global population screening. Furthermore, we aimed to assess the difference between using HLA SNPs and HLA alleles to predict type 1 diabetes risk, to determine whether SNPs serve as adequate proxies for HLA alleles. SNP genotyping is currently more affordable, globally more accessible and simpler to perform than HLA typing, making it a more practical option for large-scale screening. Our findings suggest that the combined T1D GRS_HLA_ using SNPs may enable consistently high-risk prediction across populations.

## Methods

### Participants

Unrelated individuals were assembled through the Type 1 Diabetes Genetics Consortium (T1DGC) for use in genetic studies with the custom fine-mapping ImmunoChip genotyping array [29]. The dataset included 16,198 individuals with type 1 diabetes and 25,491 control individuals, collected mainly in the USA and Europe (electronic supplementary material [ESM] Table 1). Supergroups of genetic ancestry (EUR, AFR, AMR and EAS) were defined based on *k*-means clustering [21]. The FIN population was defined within the EUR supergroup using *k*-means clustering. Principal components (PCs) for clustering were derived by projecting samples onto the 1000 Genomes Project Phase 3 reference panel using PLINK version 1.9 [30]. The South Asian (SAS) ancestry group was separated from the AMR supergroup using multi-dimensional scaling (MDS) analysis implemented in KING version 2.3.2 [31]. Together, the clustering resulted in six genetic ancestry groups (EUR, AFR, AMR, FIN, SAS, EAS). To control for population structure, PCs were generated within each group using PLINK version 1.9 [30] by performing principal component analysis (PCA) in unrelated control individuals, and projecting the individuals with type 1 diabetes onto control individuals. In addition, samples from all genetic ancestry groups (ALL) were combined, and PCs were generated by projection of all samples onto the 1000 Genomes Phase 3 reference panel. For PCA, we excluded regions of high linkage disequilibrium [32], pruned for short-range linkage disequilibrium (*r*^2^>0.2 in 50 kb windows), and removed SNPs with a minor allele frequency ≤0.05. Individuals assigned to SAS and EAS were excluded from the primary analyses due to small sample size.

### Type 1 diabetes diagnosis: eligibility criteria

Diagnosis of type 1 diabetes is critical in determining the precision of GRS. In this analysis, the type 1 diabetes individuals were obtained from two primary sources, the T1DGC and the SEARCH studies [16, 19]. Exclusion of type 1 diabetes (control individuals) was based upon the absence of a diagnosis and insulin use. In the T1DGC study, the eligibility criteria were (1) diagnosis before 35 years of age; (2) use of insulin within 6 months of diagnosis; and (3) continuous use of insulin without stopping for 6 months or more. If any question of diagnosis occurred, a clinical committee evaluated available records for decision on inclusion. In the SEARCH study, a type 1 diabetes individual was defined as an individual under 20 years of age with physician-diagnosed diabetes, confirmed by medical records review or provider reports, an in-person visit or follow-up laboratory testing (including C-peptide).

### Genotyping

Genotype data in the HLA region on human chromosome 6 (28–34 Mbp) were extracted from the ImmunoChip panel, with quality control performed as previously described [21]. Briefly, DNA samples were genotyped on the Illumina ImmunoChip, raw genotyping files were assembled, and genotype clusters were generated using the Illumina GeneTrain2 algorithm [19]. Variant filters were applied to (1) re-annotate positions by aligning probe sequences to GRCh37 (hg19) and remove any variants with <100% match or multiple matches at different positions in the genome; (2) remove variants with call rates <98%; (3) remove variants with any discordance between duplicate or monozygotic twin samples, as confirmed by genotype-inferred relationships; and (4) remove variants with Mendelian inconsistencies in >1% of the informative trios or parent–offspring pairs, based on genotype-inferred relationships. X chromosome heterozygosity and Y chromosome missingness was used to identify and exclude participants with apparent sex chromosome anomalies or inconsistencies with the reported sex using KING version 2.1.3 [31]. Samples with a genotype call rate <98% were removed. Variants with genotype frequencies deviating from Hardy–Weinberg equilibrium (*p*<5 × 10^−5^) in unrelated EUR-ancestry control individuals were excluded before imputation. All DNA samples were collected after approval from relevant institutional research ethics committees, and appropriate informed consent was obtained from all participants and families.

### HLA imputation

HLA imputation was performed using HLA-TAPAS [33] implemented on the University of Michigan imputation server (https://imputationserver.sph.umich.edu/index.html). SNPs in the HLA region (28–34 Mbp) were used to predict classical alleles for HLA class I genes (HLA-*A*, -*B* and -*C*) and HLA class II genes (*HLA-DQA1*, -*DQB1*, -*DRB1*, -*DPA1* and -*DPB1*) with two-field resolution. For quality control after imputation, any variant with an imputation accuracy of *r*^2^≤0.5 and minor allele frequency ≤0.005 was removed from further analysis. All coordinates are reported in GRCh37.

### Association analysis with type 1 diabetes to select SNPs and HLA alleles for the T1D GRS_HLA_

SNPs and HLA class I and II alleles were analysed for association with type 1 diabetes within each ancestry group (AFR, AMR, EUR, FIN) and using the combined dataset (ALL) from these groups. Logistic regression models were implemented in PLINK version 1.9, adjusting for five ancestry-specific PCs and using a minor allele count ≥20 as the filter.

Conditional analyses were performed on SNPs and HLA alleles to identify statistically independent contributors to type 1 diabetes in each group (AFR, AMR, EUR, FIN and ALL) separately. A list of conditionally independent variants was developed by (1) including the most associated variant in the logistic regression model; and (2) progressively incorporating the next most significant variants until reaching the last variant that surpassed the significance threshold. For each ancestry group, statistical significance was determined using a Bonferroni-corrected *p* value threshold (α = 0.05) correcting for the total number of SNPs (*p*<3.5 × 10^−6^) and HLA alleles (*p*<3.6 × 10^−4^). For the combined dataset (ALL), statistical significance for SNPs was *p*<3.4 × 10^−6^ and that for HLA alleles was *p*<2.8 × 10^−4^.

### HLA-focused T1D GRS_HLA_

Within each ancestry group (AFR, AMR, EUR, FIN) and across ancestry groups (ALL) HLA-focused T1D GRS_HLA_ were developed using two separate genetic markers: SNPs (T1D GRS_HLA-SNP_) and classical HLA alleles (T1D GRS_HLA-allele_). To capture the genetic contribution of the HLA region to type 1 diabetes, within each group (AFR, AMR, EUR, FIN and ALL), SNPs identified from conditional analysis were selected to create T1D GRS_HLA-SNP-AFR_, T1D GRS_HLA-SNP-AMR_, T1D GRS_HLA-SNP-EUR_, T1D GRS_HLA-SNP-FIN_ and T1D GRS_HLA-SNP-ALL_, and HLA alleles identified from conditional analyses were used to create T1D GRS_HLA-allele-AFR_, T1D GRS_HLA-allele-AMR_, T1D GRS_HLA-allele-EUR_, T1D GRS_HLA-allele-FIN_ and T1D GRS_HLA-allele-ALL_. In total, five T1D GRS_HLA_ models were developed for each type of genetic marker. ESM Tables 2–6 provide lists of SNPs included in each T1D GRS_HLA-SNP_ model (including information on effect allele, allele frequency and regression coefficient), and ESM Tables 7–11 provide lists of HLA alleles included in each T1D GRS_HLA-allele_ model. The regression coefficients for each SNP (or classical HLA allele) included in the GRS_HLA_ model were used as weights for the individual SNPs (or HLA alleles). A GRS_HLA_ for each individual was calculated by summing the allele counts multiplied by their respective weight using KING version 2.3.2.

### Transferability of T1D GRS_HLA_ in SNPs and HLA alleles

Each T1D GRS_HLA_ model (AFR, AMR, EUR, FIN and ALL) constructed from SNPs and HLA alleles was applied to individuals in all ancestry groups (AFR, AMR, EUR, FIN). All participants in each ancestry cohort had five T1D GRS_HLA-SNP_ values and five T1D GRS_HLA-allele_ values. To determine the equivalence of T1D GRS_HLA_ risk prediction across ancestry-specific T1D GRS_HLA-SNP_ and T1D GRS_HLA-allele_ models, receiver operating characteristic (ROC) AUC values were computed using the pROC R package [34]. The test to compare two AUC values used the DeLong method to determine the equivalence of AUC values [35, 36].

## Results

A total of 41,689 samples and 13,695 SNPs genotyped in the HLA region were included in the study, comprising 16,198 individuals with type 1 diabetes and 25,491 individuals without type 1 diabetes (control individuals). For each ancestry group, 425 HLA alleles were imputed at two-field resolution using a multi-ethnic HLA reference panel (HLA-TAPAS). SNPs were projected onto 1000 Genomes Project reference populations, and individuals were assigned to EUR (*N*=33,601), AFR (*N*=3877), AMR (*N*=1084) and FIN (*N*=2804) genetic ancestry groups. Individuals assigned to SAS (*N*=179) and EAS (*N*=144) ancestry groups were excluded from the primary analyses due to small sample size, limiting the final sample size to 41,366 participants.

### Conditionally independent HLA region variants associated with type 1 diabetes risk

We tested SNPs and classical HLA alleles within the MHC region to identify variants that have independent effects on type 1 diabetes risk. The SNP most significantly associated with type 1 diabetes, across all ancestries and combined data, was rs9273363 (OR_AFR_=5.56, *p*_AFR_=1.04 × 10^−133^; OR_AMR_=3.72, *p*_AMR_=1.09 × 10^−38^; OR_EUR_=4.81, *p*_EUR_=8.36 × 10^−1464^; OR_FIN_=3.64, *p*_FIN_=1.19 × 10^−88^; OR_ALL_=4.76, *p*_ALL_=3.15 × 10^−1738^). When we tested HLA alleles for association, the most significant association with risk in AFR and AMR ancestry was with HLA-DQA1*03:01 (OR_AFR_=5.45, *p*_AFR_=9.28 × 10^−116^; OR_AMR_=2.91, *p*_AMR_=2.44 × 10^−21^), while HLA-DQB1*03:02 was most strongly associated with type 1 diabetes risk in EUR and FIN ancestry (OR_EUR_=5.33, *p*_EUR_=5.08 × 10^−1145^; OR_FIN_=3.91, *p*_FIN_=8.76 × 10^−76^). The most significantly associated HLA allele in the combined ancestry data was HLA-DQB1*03:02 (OR_ALL_=5.13, *p*_ALL_=5.28 × 10^−1314^). In addition to identifying the most significant variant in each population, we conducted a stepwise conditional analysis to identify additional independently associated type 1 diabetes risk variants. The number of conditionally independent SNPs and HLA alleles is provided below in the GRS models, and the details, including allele weights and allele frequencies, are provided in ESM Tables 2–6 and ESM Tables 7–11, respectively.

### HLA-focused T1D GRS_HLA_

**Fig. 1:**
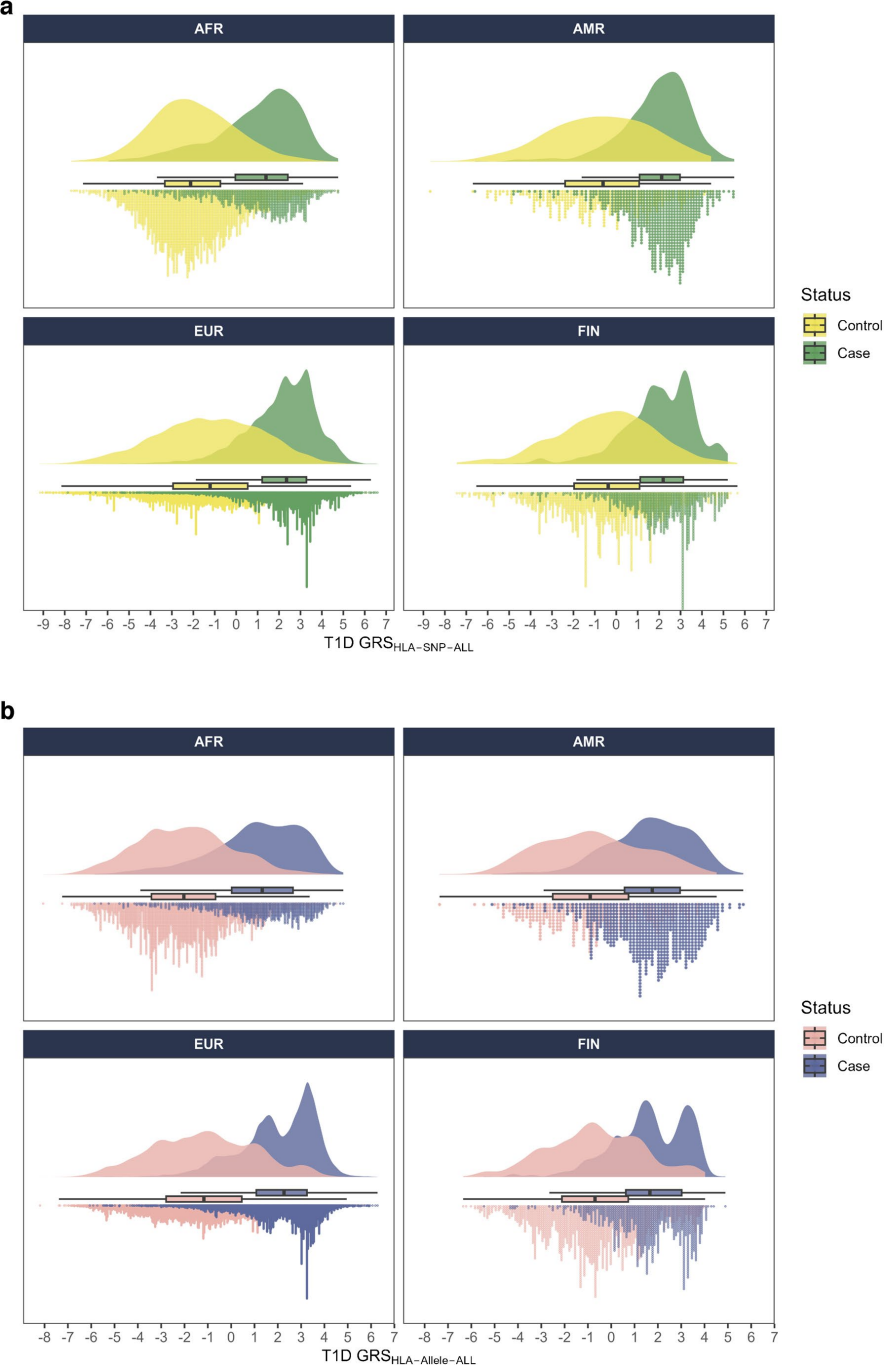
Raincloud plots of T1D GRS_HLA_ using combined ancestry data (ALL) SNPs (**a**) or HLA alleles (**b**) in four ancestry groups. The *x* axis represents the HLA-focused T1D GRS_HLA_ by status: type 1 diabetes: green in (**a**) and blue in (**b**) vs control (no type 1 diabetes); yellow in (**a**) and pink in (**b**) in each ancestry group. The dots below the box plot represent the individual scores, while the distribution is plotted above the box plot. The box plot (median, IQR and range) is shown between the upper and lower distributions

Genetic risk scores for type 1 diabetes derived from the HLA region (T1D GRS_HLA_) were calculated using SNPs (T1D GRS_HLA-SNP_) and HLA alleles (T1D GRS_HLA-allele_) across four ancestry populations (AFR, AMR, EUR, FIN) and combined data (ALL) using SNPs and imputed HLA alleles that were independently associated with type 1 diabetes. Of the four ancestry groups, EUR had the largest population size and the EUR-derived T1D GRS_HLA_ models contained the most SNPs (*n*=38) and HLA alleles (*n*=40), while other GRS_HLA_ models had less than 10 SNPs and HLA alleles with conditional independence of association with type 1 diabetes. The T1D GRS_HLA_ developed in AFR consists of 5 SNPs and 6 HLA alleles, in AMR of 3 SNPs and 5 HLA alleles, and in FIN of 6 SNPs and 8 HLA alleles (ESM Tables 3–6 and ESM Tables 8–11). The model constructed from combined data (ALL) was dominated by those SNPs and HLA alleles from the EUR population, and the T1D GRS_HLA_ models contained 36 SNPs and 41 HLA alleles (ESM Table 2 and ESM Table 7). Each T1D GRS_HLA_ model (AFR, AMR, EUR, FIN and ALL) was applied to all ancestry groups (AFR, AMR, EUR, FIN). We plotted the distribution of each T1D GRS_HLA_ in all ancestry groups separating T1D individuals and control individuals. The distribution of T1D GRS_HLA_ within all populations exhibited significant overlap in those with type 1 diabetes and those without type 1 diabetes, whether using SNPs (Fig. 1a) or imputed HLA alleles (Fig. 1b). The bimodal distribution observed in the EUR and FIN populations can be explained by the presence of a substantial number of individuals carrying high-risk HLA haplotypes (HLA-DR3 and/or DR4), who exhibit the highest T1D GRS values. ESM Fig. 1 shows the distribution of T1D GRS_HLA-allele-ALL_, applied across all ancestry groups (AFR, AMR, EUR, FIN) and stratified by individuals carrying high-risk HLA haplotypes (HLA-DR3 and/or DR4) and control individuals.

### Prediction and transferability of type 1 diabetes using ROC curve analysis

**Fig. 2:**
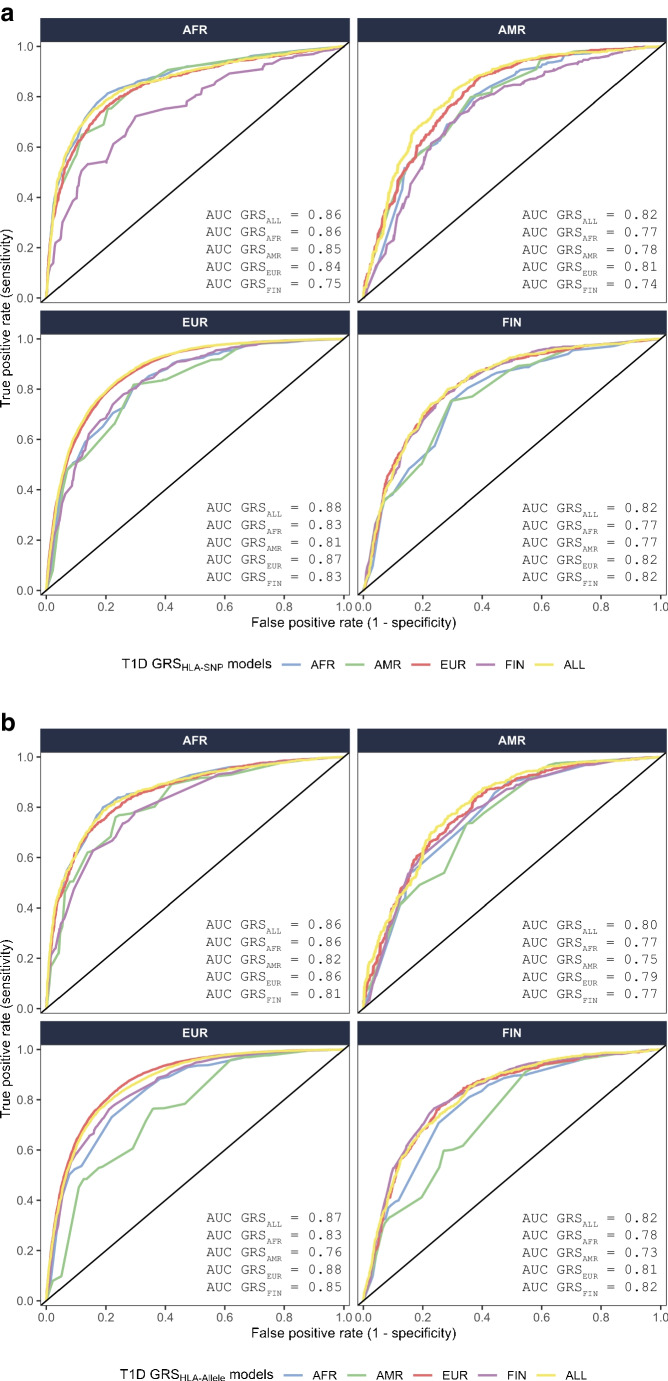
AUC from ROC analyses of T1D GRS_HLA_ based on HLA SNPs (**a**) or HLA alleles (**b**) in four ancestry groups. Each T1D GRS_HLA_ model is colour-coded based upon the ancestry from which it was derived (AFR, blue; AMR, green; EUR, red; FIN, purple; ALL, yellow). The AUC values for each model are presented in the lower right corner for each ancestry group

The accuracy of prediction of type 1 diabetes risk (defined by ROC AUC) using SNPs (Fig. 2a and ESM Table 12) was uniformly high, ranging from 0.74 (T1D GRS_HLA-SNP-FIN_ applied to AMR) to 0.88 (T1D GRS_HLA-SNP-ALL_ applied to EUR). Similarly, the ROC AUC using HLA alleles (Fig. 2b and ESM Table 13) ranged from 0.73 (T1D GRS_HLA-allele-AMR_ applied to FIN) to 0.88 (T1D GRS_HLA-allele-EUR_ to EUR). The T1D GRS_HLA_ model based upon combined data (T1D GRS_HLA-SNP-ALL_ and T1D GRS_HLA-allele-ALL_) performed equivalently to the best individual ancestry-derived models across groups, whether using SNPs or HLA alleles. There were no significant differences in model performance, whether using SNPs or imputed HLA alleles, for any comparison.

To evaluate the transferability of scores, we compared ancestry-derived T1D GRS_HLA_ scores with T1D GRS_HLA-ALL_ scores within each ancestry group (Tables 1 and 2). In the AFR ancestry group, the performance of T1D GRS_HLA-SNP-ALL_ did not differ significantly from that of T1D GRS_HLA-SNP-AFR_ (AUC_ALL_=0.86 vs AUC_AFR_=0.86, *p*=0.11). Similarly, in the FIN ancestry group, the performance of T1D GRS_HLA-SNP-ALL_ did not differ significantly from that of T1D GRS_HLA-SNP-FIN_ (AUC_ALL_=0.82 vs AUC_FIN_=0.82, *p*=0.79). In contrast, T1D GRS_HLA-SNP-ALL_ performed significantly better in the AMR ancestry group than the AMR-specific T1D GRS_HLA_ (AUC_ALL_=0.82 vs AUC_AMR_=0.78, *p*=7.86 × 10^−6^) and the EUR-specific T1D GRS_HLA_ (AUC_ALL_=0.88 vs AUC_EUR_=0.87, *p*=4.73 × 10^−6^).

**Table 1:** Performance of HLA-focused T1D GRS_HLA-SNP-ALL_ compared with ancestry-specific T1D GRS_HLA_ in each ancestry

Ancestry	GRS_HLA-SNP_	GRS_HLA-SNP-ALL_	AUC_HLA-SNP_	AUC_HLA-SNP-ALL_	*p* value
AFR	AFR	ALL	0.86	0.86	0.11
AMR	AMR	ALL	0.78	0.82	7.86 × 10^−6^
EUR	EUR	ALL	0.87	0.88	4.73 × 10^−6^
FIN	FIN	ALL	0.82	0.82	0.79

*p* values were calculated using the DeLong test [35]

GRS_HLA-SNP_, T1D GRS_HLA_ constructed using individual ancestry groups (AFR, AMR, EUR or FIN); GRS_HLA-SNP-ALL_, T1D GRS_HLA_ constructed using combined ancestry data (ALL); AUC_HLA-SNP_, AUC for T1D GRS_HLA-SNP_ applied in each ancestry group; AUC_HLA-SNP-ALL_, AUC for T1D GRS_HLA-SNP-ALL_ applied in each ancestry group

**Table 2:** Performance of HLA-focused T1D GRS_HLA-allele-ALL_ compared with ancestry-specific T1D GRS_HLA_ in each ancestry

Ancestry	GRS_HLA-allele_	GRS_HLA-allele-ALL_	AUC_HLA-allele_	AUC_HLA-allele-ALL_	*p* value
AFR	AFR	ALL	0.86	0.86	0.72
AMR	AMR	ALL	0.75	0.80	2.30 × 10^−6^
EUR	EUR	ALL	0.88	0.87	3.50 × 10^−22^
FIN	FIN	ALL	0.82	0.82	0.19

*p* values were calculated using the DeLong test [35]

GRS_HLA-allele_, T1D GRS_HLA_ constructed using individual ancestry groups (AFR, AMR, EUR or FIN); GRS_HLA-allele-ALL_, T1D GRS_HLA_ constructed using combined ancestry data (ALL); AUC_HLA-allele_, AUC for T1D GRS_HLA-allele_ applied in each ancestry group; AUC_HLA-allele-ALL_, AUC for T1D GRS_HLA-allele-ALL_ applied in each ancestry group

### Type 1 diabetes risk prediction in an independent cohort and the effect of non-HLA SNPs

In order to address two limitations of cross-ancestry group comparisons, we included non-HLA-region SNPs [21] in the T1D GRS_HLA-SNP-ALL_ model and recalculated the T1D GRS in each ancestry, and also conducted a validation study in a population of diverse ancestry. Incorporating non-HLA SNPs in the T1D GRS score improved prediction in all groups. The AUC in the AFR group increased from 0.86 to 0.88, and those in the AMR and FIN groups increased from 0.82 to 0.85 and from 0.82 to 0.84, respectively. In the EUR group, the AUC increased from 0.88 to 0.91. Together, these results suggest that inclusion of non-HLA SNPs increases the predictive accuracy of the T1D GRS. In a larger, genetically diverse validation cohort (510 individuals with type 1 diabetes, 6342 control individuals; 30% AFR, 18% AMR, 11% EAS, 41% EUR), using 23 HLA-region SNPs yielded an AUC of 0.806. Inclusion of 67 non-HLA-region SNPs in addition to the HLA-region SNPs resulted in only a slight increase in predictive performance (AUC=0.810). Thus, the T1D GRS that included all SNPs was validated with a high AUC (approximately 0.80), even though non-HLA SNPs did not significantly improve the AUC beyond that achieved by HLA SNPs alone.

## Discussion

In this study, we constructed T1D GRS_HLA_ in various ancestry groups, as well as combining data across populations. To develop GRS models, we identified independently associated SNPs, HLA class I alleles, and HLA class II alleles in admixed African, admixed American, European and Finnish groups and across all ancestries. We applied each T1D GRS_HLA_ model to the four ancestry groups. Our results suggest that T1D GRS_HLA_ derived from combined ancestry data (ALL) performed equivalently to or better than ancestry-specific T1D GRS_HLA_ models defined in each ancestry group.

Each T1D GRS_HLA_ model included various numbers of conditionally independent HLA-region SNPs associated with type 1 diabetes. The SNP most significantly associated with type 1 diabetes, across all ancestries and combined data, was rs9273363. This SNP has been identified as the most strongly associated with type 1 diabetes risk, and tags HLA-DQB1*03:02 in European ancestry populations. As expected, HLA-DQB1*03:02 was most strongly associated with type 1 diabetes risk in the EUR and FIN groups, while the most strongly associated HLA class II allele in the AFR and AMR groups was HLA-DQA1*03:01, which aligns with our previous findings from recent type 1 diabetes genome-wide association study [24]. Given the much larger EUR sample size in the genome-wide association study (and in the current HLA-focused study), the analysis of samples from all ancestry groups also identified HLA-DQB1*03:02 as the HLA allele most strongly associated with type 1 diabetes risk.

It is well known that the HLA region has undergone selective pressure [37], leading to changes in allele frequency across populations. Although many of the SNPs and HLA alleles associated with type 1 diabetes risk with respect to the HLA region are shared across ancestries, the size of the effect on risk can vary dramatically. In this study, the effect of HLA-DQB1*03:02 and the tagging SNP rs9273363 on type 1 diabetes risk varied by ancestry. For rs9273363, the effect size in the AFR group (OR=5.56) is larger than that in the AMR (OR=3.72), EUR (OR=4.81) and FIN (OR=3.64) groups. In contrast, the effect size for HLA-DQB1*03:02 allele tagged by this SNP differs among the AFR (OR=1.68; the HLA-DQA1*03:01 allele is most strongly associated with type 1 diabetes risk, with effect OR=5.45), AMR (not significant in this group), EUR (OR=5.33) and FIN (OR=3.91) groups. These results highlight the complexity of the HLA associations with type 1 diabetes and the need to develop scores that include ancestry-informed risk prediction.

The methodology of GRS is being adopted in the clinic and in population screening to reduce complexity into a single metric, similar to any clinical laboratory test (e.g. HDL-cholesterol), to enable comparison with a standard reference range. A major limitation has been the reliance on genetic data from studies of European ancestry [17]. In type 1 diabetes, the majority of studies have adopted T1D GRS [27, 28] that have been generated using European ancestry data. These GRS perform well to distinguish type 1 diabetes from type 2 diabetes, predict progression to insulin deficiency and improve newborn screening. In addition, the T1D GRS1 has been shown to discriminate type 1 diabetes from type 2 diabetes and maturity-onset diabetes of the young (MODY) [38]. However, there is a critical need to include non-European populations in the development of more accurate GRS, and to create T1D GRS models that incorporate diverse ancestry populations. Recent applications of T1D GRS [23, 39–41] and methods development by two consortia (eMERGE and PRIMED) are beginning to address these gaps.

In the present paper, we have recalculated weights for associated SNPs within each ancestry group and across all individuals. This is in contrast with existing methods [27, 28], in which weights are driven by European populations. There are increasing efforts to assess the performance of T1D GRS1 and T1D GRS2, which were developed in EUR-ancestry populations, when applied to other populations. Recent analysis of an Indian T1D GRS comprising 67 SNPs discriminated type 1 diabetes individuals from control individuals in an Indian population with an AUC of 0.83, which is lower than that for the EUR T1D GRS in EUR populations (AUC=0.92) [42]. An updated score (T1D GRS2x) was used in the genetically diverse ‘All of Us’ Research Program cohort [43], and was highly predictive (multi-ancestry AUC=0.86) but lower than in EUR (AUC=0.895). The utility of multi-ancestry T1D GRS and their development across diverse ancestries show promise, but increased sample size and use of more diverse ancestry groups are required to provide improvements beyond those of European ancestry.

This study has several strengths and limitations. Strengths of this study include the use of multi-ancestry cohorts, focus on the most important genomic region for type 1 diabetes risk (the HLA region), use of imputation to increase the number of SNPs and HLA alleles (using HLA-TAPAS), and comparison of ancestry-specific SNP- and HLA allele-based GRS. However, there are some limitations, including the smaller number of individuals in under-represented ancestry-diverse populations (AFR and AMR), and the exclusion of potentially informative populations due to extremely small sample size (EAS and SAS). In addition, not all HLA alleles could be imputed in all populations (e.g. HLA DQB1*02:02). An additional limitation is that there is an overlap between the training data and the testing data that may affect interpretation of performance. These limitations highlight the need to expand non-European cohorts to better assess the prediction of ancestry-specific GRS for all diseases, including type 1 diabetes.

In summary, our data suggest that T1D GRS_HLA_ derived from SNPs perform equivalently to T1D GRS_HLA_ derived from HLA alleles across ancestries. The T1D GRS_HLA_ model derived from one ancestry is not uniformly predictive in other ancestries. Greater sample size in non-European ancestry populations is needed to develop more accurate non-EUR T1D GRS_HLA_. In addition, our results suggest that the T1D GRS_HLA-ALL_ constructed from combined ancestry data performed equally well or better than ancestry-specific scores in each ancestry. While we do not have a clear explanation, we believe that T1D GRS_HLA-ALL_ performed better than the individual ancestry scores because there is more power to detect type 1 diabetes-associated SNPs with greater sample size. In addition, we included individuals of diverse ancestry, allowing us to tag ancestry-specific type 1 diabetes risk HLA alleles and haplotypes, compared with using European-ancestry SNPs only.

## Supplementary Information

Below is the link to the electronic supplementary material.

ESM Figure (PDF 124 KB)

ESM Tables (XLSX 40.1 KB)

## Data Availability

Summary statistics are available in dbGaP (https://dbgap.ncbi.nlm.nih.gov/home) under accession number pha002468.v2.p1, and the Accelerating Medicines Partnership Common Metabolic Diseases (AMP CMD) Knowledge Portal (https://hugeamp.org/).

## Abbreviations

AFR: Admixed African (ancestry group)
ALL: All ancestries
AMR: Admixed American (ancestry group)
EAS: East Asian
EUR: European (ancestry group)
FIN: Finnish (ancestry group)
GRS: Genetic risk score(s)
PCA: Principal component analysis
PC: Principal component
ROC: Receiver operating characteristic
SAS: South Asian
SEARCH: SEARCH for Diabetes in Youth
T1D GRS: Type 1 diabetes genetic risk score
T1DGC: Type 1 Diabetes Genetics Consortium

## Code availability

Code used to generate results is available at https://github.com/damichalek/T1DGC_GRS_HLA.
