# Supplementary material for: HLA-focused type 1 diabetes genetic risk prediction in populations of diverse ancestry
Source: Diabetologia. 2025 Oct 2;69(1):146–56. doi: 10.1007/s00125-025-06563-8 (PMC12685975; doi:10.1007/s00125-025-06563-8)
Supplement: Supplementary file 1 — ESM Figure (PDF 124 KB) [file 125_2025_6563_MOESM1_ESM.pdf]

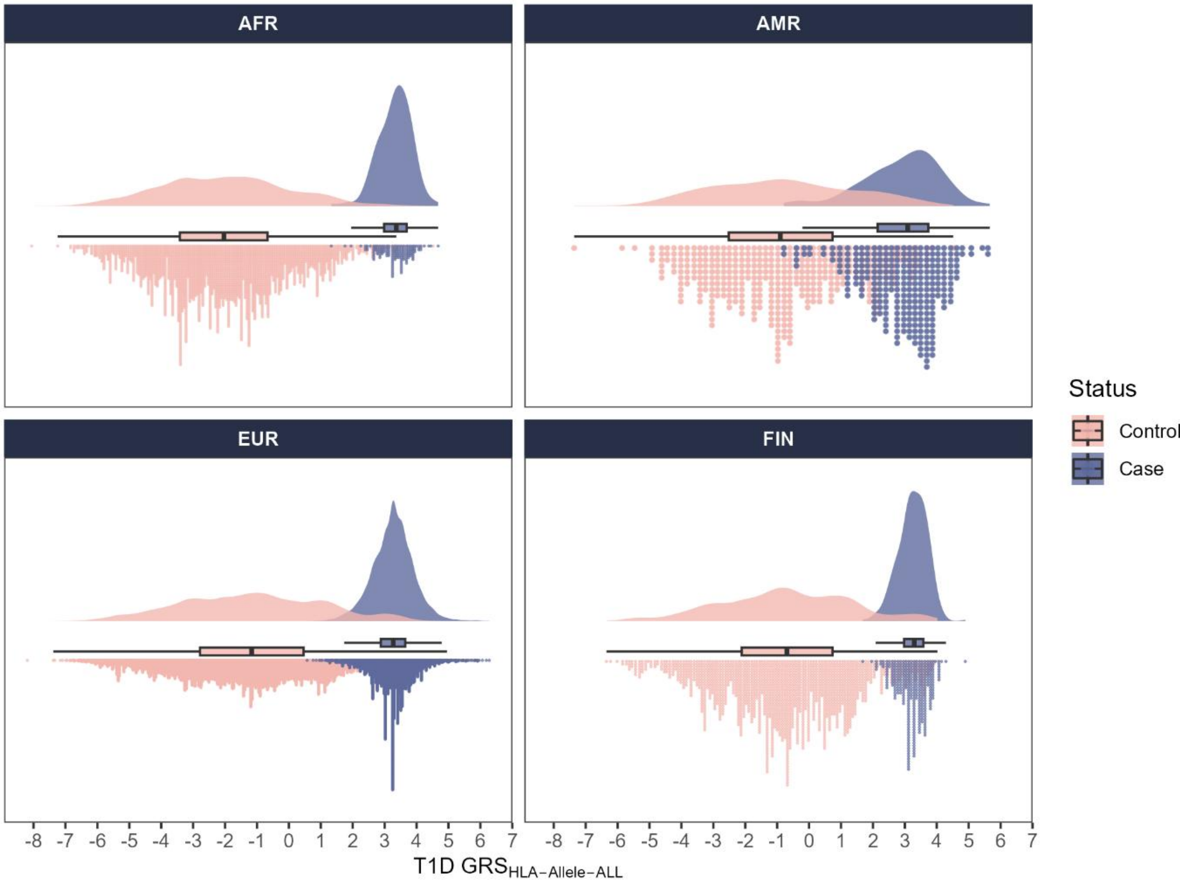

ESM Figure 1. Raincloud plots of HLA-focused type 1 diabetes genetic risk score, T1D $GRS_{HLA\text{-}Allele\text{-}ALL}$, across four ancestry groups. T1D $GRS_{HLA\text{-}Allele\text{-}ALL}$ was derived using combined ancestry data (ALL) and then applied to each ancestry group separately. Case subjects were stratified to include only individuals carrying high-risk HLA haplotypes (e.g., HLA-DR3 and/or -DR4). The x-axis shows T1D $GRS_{HLA\text{-}Allele\text{-}ALL}$ for type 1 diabetes cases with HLA-DR3 and/or -DR4 haplotypes (blue) and controls without type 1 diabetes (pink) in each ancestry group. Each plot displays a box plot (median, interquartile range, and range). The dots below the box plot represent the individual scores, and the density distribution is plotted above the box plot.
